# Supplementary material for: Functional characterisation of phagocytes in the Pacific oyster Crassostrea gigas
Source: PeerJ. 2016 Dec 14;4:e2590. doi: 10.7717/peerj.2590 (PMC5160923; doi:10.7717/peerj.2590)
Supplement: Supplemental Information 1 — All the experiments were performed on a flow cytometer BD FACS Aria II. Error bars were generated as the standard error of the mean from five or six replicates. For each samples, at least 10,000 experimental cells were acquired. [file peerj-04-2590-s001.docx]

Functional characterization of phagocytes in the Pacific oyster *Crassostrea gigas*

Shuai Jiang^a^, Zhihao Jia^a^, Tao Zhang^a^, Lingling Wang^a^, Jinsheng Sun^c^, Limei Qiu^a^, Linsheng Song^b,^ *

^a^ Key laboratory of Experimental Marine Biology, Institute of Oceanology, Chinese Academy of Sciences, Qingdao, 266071, China.

^b^ Key Laboratory of Mariculture & Stock enhancement in North China’s Sea, Ministry of Agriculture, Dalian Ocean University, Dalian 116023, China.

^c^ Tianjin Key Laboratory of Animal and Plant Resistance, Tianjin, 300387, China.

* Address correspondence to: Linsheng Song

Dalian Ocean University, No. 52 Heishijiao street, Dalian 116023, China.

Tel: +86 411 84763173; Fax: +86 411 84763306

Email address: [lshsong@dlou.edu.cn](mailto:lshsong@dlou.edu.cn)

**RAW DATA**

Fig. 1. (B) The percentages of haemocyte subpopulations were calculated by flow cytometry.

| Haemocytes | Sample 1 | Sample 2 | Sample 3 | Sample 4 | Sample 5 | Sample 6 |
| --- | --- | --- | --- | --- | --- | --- |
| agranulocytes (%) | 45.1 | 43.0 | 49.3 | 53.1 | 45.7 | 40.8 |
| granulocytes (%) | 35.7 | 28.5 | 26.9 | 32.3 | 33.4 | 31.5 |
| semigranulocytes (%) | 19.1 | 22.3 | 18.6 | 20.5 | 18.7 | 18.5 |

Fig. 3. The phagocytic percentages of total haemocytes, granulocytes and semigranulocytes were calculated by flow cytometry. TH, G and SG are short for total haemocytes, granulocytes and semigranulocytes respectively, RGD is short for Arg-Gly-Asp tripeptide.

(B) The phagocytic percentages of haemocytes towards *E. coli*.

| Haemocytes | Sample 1 | Sample 2 | Sample 3 | Sample 4 | Sample 5 | Sample 6 |
| --- | --- | --- | --- | --- | --- | --- |
| TH (%) | 24.5 | 26.7 | 11.3 | 36.1 | 24.9 | 25.1 |
| TH + RGD (%) | 8.4 | 13.6 | 10.2 | 14.5 | 14.1 | 23.5 |
| G (%) | 21.3 | 35.7 | 36.3 | 40.6 | 39.5 | 58.6 |
| G + RGD (%) | 23.9 | 12.1 | 25.5 | 24.7 | 33.8 | 24.4 |
| SG (%) | 18.0 | 18.3 | 29.5 | 19.8 | 18.9 | 10.3 |
| SG + RGD (%) | 20.3 | 10.1 | 4.5 | 6.2 | 10.5 | 9.8 |

(D) The phagocytic percentages of haemocytes towards *V. splendidus*.

| Haemocytes | Sample 1 | Sample 2 | Sample 3 | Sample 4 | Sample 5 | Sample 6 |
| --- | --- | --- | --- | --- | --- | --- |
| TH (%) | 10.8 | 8.6 | 8.9 | 8.1 | 8.5 | 4.5 |
| TH + RGD (%) | 4.1 | 4.2 | 5.4 | 5.3 | 5.9 | 4.8 |
| G (%) | 14.4 | 10.9 | 7.8 | 6.9 | 9.5 | 9.1 |
| G + RGD (%) | 5.8 | 8.4 | 6.2 | 6.7 | 5.1 | 5.9 |
| SG (%) | 5.5 | 6.9 | 6.2 | 8.1 | 7.1 | 10.8 |
| SG + RGD (%) | 3.6 | 4.1 | 4.3 | 3.6 | 3.2 | 4.3 |

(F) The phagocytic percentages of haemocytes towards *S. aureus*.

| Haemocytes | Sample 1 | Sample 2 | Sample 3 | Sample 4 | Sample 5 | Sample 6 |
| --- | --- | --- | --- | --- | --- | --- |
| TH (%) | 26.1 | 13.9 | 15.8 | 10.1 | 13.4 | 9.1 |
| TH + RGD (%) | 6.1 | 7.4 | 2.7 | 7.6 | 13.2 | 11.0 |
| G (%) | 36.7 | 24.1 | 23.9 | 14.6 | 20.7 | 24.3 |
| G + RGD (%) | 20.2 | 14.4 | 13.7 | 13.1 | 14.2 | 10.1 |
| SG (%) | 10.9 | 9.6 | 5.5 | 10.1 | 16.1 | 9.8 |
| SG + RGD (%) | 3.1 | 5.4 | 4.9 | 9.9 | 4.8 | 3.5 |

Fig. 4. The relative phagocytic activities of total haemocytes, granulocytes and semigranulocytes after carbohydrate incubation were calculated by flow cytometry. TH, G and SG are short for total haemocytes, granulocytes and semigranulocytes respectively, Glu, Fuc, Man, Lac and GlcNAc are short for glucose, fucose, mannose, lactose and N-acetylglucosamine respectively.

(A) The relative phagocytic activities of total haemocytes towards *V. splendidus.*

| Haemocytes | Sample 1 | Sample 2 | Sample 3 | Sample 4 | Sample 5 | Sample 6 |
| --- | --- | --- | --- | --- | --- | --- |
| TH (%) | 103.4 | 93.7 | 97.2 | 93.6 | 110.1 | 103.2 |
| TH + Glu (%) | 95.6 | 90.1 | 84.8 | 96 | 104.7 | 101.8 |
| TH + Fuc (%) | 84.2 | 61.6 | 33.6 | 22.5 | 51.4 | 97.1 |
| TH + Man (%) | 98.2 | 85.5 | 54.4 | 81.8 | 31.3 | 51.2 |
| TH + Lac (%) | 41.3 | 79.2 | 59.4 | 64.7 | 94.1 | 92.6 |
| TH + GlcNAc (%) | 40.5 | 42.9 | 92.2 | 66.5 | 96.3 | 49.4 |

(B) The relative phagocytic activities of granulocytes towards *V. splendidus.*

| Haemocytes | Sample 1 | Sample 2 | Sample 3 | Sample 4 | Sample 5 | Sample 6 |
| --- | --- | --- | --- | --- | --- | --- |
| G (%) | 126.2 | 96.7 | 78.3 | 115.4 | 87.5 | 98.1 |
| G + Glu (%) | 113.5 | 97.6 | 75.7 | 98.3 | 106.6 | 82.4 |
| G + Fuc (%) | 70.5 | 48.3 | 98.9 | 69.7 | 76.1 | 77.2 |
| G + Man (%) | 77.2 | 88.1 | 93.6 | 90.4 | 69.8 | 65.6 |
| G + Lac (%) | 74.9 | 66.6 | 71.7 | 70.4 | 86.2 | 83.4 |
| G + GlcNAc (%) | 74.8 | 98.3 | 64.8 | 49.1 | 72.9 | 73.4 |

(C) The relative phagocytic activities of semigranulocytes towards *V. splendidus.*

| Haemocytes | Sample 1 | Sample 2 | Sample 3 | Sample 4 | Sample 5 | Sample 6 |
| --- | --- | --- | --- | --- | --- | --- |
| SG (%) | 82.9 | 80.6 | 103.5 | 118.4 | 105.6 | 108.5 |
| SG + Glu (%) | 118.3 | 95.4 | 93.2 | 108.6 | 96.7 | 64.8 |
| SG + Fuc (%) | 88.6 | 76.5 | 19.7 | 86.3 | 27.8 | 29.8 |
| SG + Man (%) | 47.4 | 19.2 | 46.9 | 88.7 | 26.1 | 86.3 |
| SG + Lac (%) | 90.3 | 73.6 | 37.8 | 88.4 | 27.7 | 47.8 |
| SG + GlcNAc (%) | 81.7 | 78.3 | 27.9 | 72.6 | 26.9 | 86.4 |

(D) The relative phagocytic activities of total haemocytes towards *S. aureus.*

| Haemocytes | Sample 1 | Sample 2 | Sample 3 | Sample 4 | Sample 5 | Sample 6 |
| --- | --- | --- | --- | --- | --- | --- |
| TH (%) | 66.7 | 98.3 | 97.1 | 118.4 | 106.4 | 115.6 |
| TH + Glu (%) | 93.7 | 77.6 | 98.4 | 107.4 | 94.8 | 106.0 |
| TH + Fuc (%) | 43.2 | 65.3 | 64.8 | 99.2 | 82.5 | 69.7 |
| TH + Man (%) | 26.9 | 81.6 | 78.2 | 84.3 | 58.3 | 34.1 |
| TH + Lac (%) | 80.3 | 99.8 | 96.7 | 104.5 | 73.6 | 107.2 |
| TH + GlcNAc (%) | 70.6 | 81.2 | 62.9 | 50.4 | 29.7 | 88.1 |

(E) The relative phagocytic activities of granulocytes towards *S. aureus.*

| Haemocytes | Sample 1 | Sample 2 | Sample 3 | Sample 4 | Sample 5 | Sample 6 |
| --- | --- | --- | --- | --- | --- | --- |
| G (%) | 128.5 | 105.2 | 86.4 | 62.6 | 107.5 | 112.1 |
| G + Glu (%) | 117.8 | 66.4 | 109.2 | 78.7 | 107.5 | 84.3 |
| G + Fuc (%) | 60.7 | 27.4 | 63.5 | 69.8 | 85.2 | 71.3 |
| G + Man (%) | 78.7 | 66.4 | 33.5 | 20.7 | 80.6 | 69.2 |
| G + Lac (%) | 62.4 | 96.5 | 117.3 | 90.8 | 106.6 | 91.7 |
| G + GlcNAc (%) | 54.5 | 21.8 | 95.7 | 32.8 | 58.6 | 75.3 |

(F) The relative phagocytic activities of semigranulocytes towards *S. aureus.*

| Haemocytes | Sample 1 | Sample 2 | Sample 3 | Sample 4 | Sample 5 | Sample 6 |
| --- | --- | --- | --- | --- | --- | --- |
| SG (%) | 85.3 | 116.8 | 119.5 | 101.4 | 67.6 | 108.7 |
| SG + Glu (%) | 69.3 | 111.6 | 107.5 | 74.8 | 93.3 | 104.2 |
| SG + Fuc (%) | 93.6 | 45.9 | 78.7 | 80.5 | 52.6 | 85.3 |
| SG + Man (%) | 59.3 | 88.9 | 64.5 | 42.7 | 75.6 | 90.1 |
| SG + Lac (%) | 108.4 | 96.9 | 107.5 | 94.3 | 78.7 | 71.6 |
| SG + GlcNAc (%) | 83.6 | 86.3 | 69.1 | 75.6 | 81.5 | 87.0 |

Fig. 5. The relative phagocytic activities of total haemocytes, granulocytes and semigranulocytes after LPS and PGN incubation were calculated by flow cytometry respectively. TH, G and SG are short for total haemocytes, granulocytes and semigranulocytes respectively, LPS and PGN are short for lipopolysaccharide and peptidylglycan respectively.

(A) The relative phagocytic activities of total haemocytes towards *V. splendidus.*

| Haemocytes | Sample 1 | Sample 2 | Sample 3 | Sample 4 | Sample 5 | Sample 6 |
| --- | --- | --- | --- | --- | --- | --- |
| TH (%) | 113.6 | 105.7 | 102.6 | 88.4 | 86.7 | 102.8 |
| TH + 0.01 mg/ml LPS (%) | 142.6 | 106.9 | 103.4 | 110.1 | 128.5 | 115.5 |
| TH + 0.1 mg/ml LPS (%) | 133.8 | 179.7 | 88.1 | 132.7 | 164.5 | 167.2 |
| TH + 0.01 mg/ml PGN (%) | 113.5 | 96.7 | 72.6 | 118.4 | 65.4 | 107.2 |
| TH + 0.1 mg/ml PGN (%) | 74.8 | 108.1 | 95.7 | 86.8 | 97.4 | 115.6 |

(B) The relative phagocytic activities of granulocytes towards *V. splendidus.*

| Haemocytes | Sample 1 | Sample 2 | Sample 3 | Sample 4 | Sample 5 | Sample 6 |
| --- | --- | --- | --- | --- | --- | --- |
| G (%) | 76.7 | 98.9 | 105.4 | 97.5 | 121.4 | 100.7 |
| G + 0.01 mg/ml LPS (%) | 110.8 | 108.9 | 125.2 | 113.7 | 111.6 | 99.1 |
| G + 0.1 mg/ml LPS (%) | 123.3 | 118.5 | 116.4 | 141.6 | 96.9 | 115.8 |
| G + 0.01 mg/ml PGN (%) | 118.2 | 95.4 | 77.5 | 81.8 | 97.2 | 105.6 |
| G + 0.1 mg/ml PGN (%) | 113.9 | 75.4 | 97.1 | 93.5 | 107.4 | 83.8 |

(C) The relative phagocytic activities of semigranulocytes towards *V. splendidus.*

| Haemocytes | Sample 1 | Sample 2 | Sample 3 | Sample 4 | Sample 5 | Sample 6 |
| --- | --- | --- | --- | --- | --- | --- |
| SG (%) | 102.8 | 85.9 | 71.7 | 98.6 | 132.2 | 110.6 |
| SG + 0.01 mg/ml LPS (%) | 134.2 | 151.8 | 122.6 | 96.9 | 113.7 | 133.8 |
| SG + 0.1 mg/ml LPS (%) | 117.8 | 172.1 | 139.7 | 163.8 | 140.4 | 187.9 |
| SG + 0.01 mg/ml PGN (%) | 112.3 | 98.6 | 84.5 | 73.8 | 91.1 | 105.2 |
| SG + 0.1 mg/ml PGN (%) | 72.7 | 108.9 | 93.4 | 85.2 | 118.4 | 96.1 |

(D) The relative phagocytic activities of total haemocytes towards *S. aureus.*

| Haemocytes | Sample 1 | Sample 2 | Sample 3 | Sample 4 | Sample 5 | Sample 6 |
| --- | --- | --- | --- | --- | --- | --- |
| TH (%) | 115.7 | 113.5 | 102.7 | 83.6 | 98.2 | 87.6 |
| TH + 0.01 mg/ml LPS (%) | 131.1 | 109.3 | 114.9 | 118.6 | 103.2 | 123.7 |
| TH + 0.1 mg/ml LPS (%) | 145.7 | 99.3 | 134.8 | 141.2 | 106.7 | 161.8 |
| TH + 0.01 mg/ml PGN (%) | 94.7 | 78.6 | 117.2 | 91.8 | 82.6 | 105.4 |
| TH + 0.1 mg/ml PGN (%) | 97.8 | 105.4 | 87.1 | 76.9 | 118.6 | 90.4 |

(E) The relative phagocytic activities of granulocytes towards *S. aureus.*

| Haemocytes | Sample 1 | Sample 2 | Sample 3 | Sample 4 | Sample 5 | Sample 6 |
| --- | --- | --- | --- | --- | --- | --- |
| G (%) | 108.2 | 87.5 | 93.6 | 105.4 | 112.7 | 94.8 |
| G + 0.01 mg/ml LPS (%) | 125.4 | 104.8 | 115.4 | 119.5 | 114.2 | 106.3 |
| G + 0.1 mg/ml LPS (%) | 154.7 | 159.5 | 119.2 | 117.5 | 108.4 | 117.1 |
| G + 0.01 mg/ml PGN (%) | 79.2 | 107.5 | 96.8 | 90.5 | 102.8 | 87.1 |
| G + 0.1 mg/ml PGN (%) | 98.4 | 80.8 | 107.6 | 95.9 | 76.2 | 106.7 |

(F) The relative phagocytic activities of semigranulocytes towards *S. aureus.*

| Haemocytes | Sample 1 | Sample 2 | Sample 3 | Sample 4 | Sample 5 | Sample 6 |
| --- | --- | --- | --- | --- | --- | --- |
| SG (%) | 98.6 | 116.3 | 104.2 | 95.8 | 82.1 | 102.6 |
| SG + 0.01 mg/ml LPS (%) | 134.7 | 120.9 | 128.6 | 117.4 | 118.5 | 96.8 |
| SG + 0.1 mg/ml LPS (%) | 126.1 | 109.6 | 162.9 | 166.5 | 131.2 | 108.7 |
| SG + 0.01 mg/ml PGN (%) | 92.7 | 87.6 | 108.4 | 72.6 | 106.8 | 104.4 |
| SG + 0.1 mg/ml PGN (%) | 90.8 | 108.6 | 97.2 | 89.8 | 103.2 | 95.4 |

Fig. 6. (B) The percentages of haemocytes gated on PE^+^/FITC^+^, PE^+^/FITC^-^ and PE^-^/FITC^+^ were calculated by flow cytometry.

Wheat germ agglutinin staining

| Microbes | Fluorescence | Sample 1 | Sample 2 | Sample 3 | Sample 4 | Sample 5 |
| --- | --- | --- | --- | --- | --- | --- |
| *E. coli* | PE^+^/FITC^+^ (%) | 20.4 | 19.7 | 31.8 | 24.6 | 12.5 |
|  | PE^+^/FITC^-^ (%) | 16.4 | 28.9 | 25.7 | 30.6 | 24.6 |
|  | PE^-^/FITC^+^ (%) | 9.7 | 17.2 | 14.8 | 11.7 | 12.6 |
| *V. splendidus* | PE^+^/FITC^+^ (%) | 17.9 | 9.8 | 12.7 | 16.4 | 10.2 |
|  | PE^+^/FITC^-^ (%) | 29.6 | 24.2 | 33.6 | 39.7 | 42.3 |
|  | PE^-^/FITC^+^ (%) | 18.9 | 12.5 | 17.4 | 21.8 | 18.1 |
| *B. subtilis* | PE^+^/FITC^+^ (%) | 25.8 | 12.7 | 15.9 | 20.6 | 19.4 |
|  | PE^+^/FITC^-^ (%) | 47.8 | 40.1 | 29.6 | 36.7 | 33.9 |
|  | PE^-^/FITC^+^ (%) | 10.3 | 9.7 | 18.4 | 13.6 | 15.8 |
| *S. aureus* | PE^+^/FITC^+^ (%) | 33.2 | 27.6 | 23.4 | 39.8 | 30.5 |
|  | PE^+^/FITC^-^ (%) | 30.6 | 38.6 | 32.7 | 27.6 | 42.1 |
|  | PE^-^/FITC^+^ (%) | 7.5 | 12.6 | 8.6 | 10.5 | 8.7 |

Peanut agglutinin staining

| Microbes | Fluorescence | Sample 1 | Sample 2 | Sample 3 | Sample 4 | Sample 5 |
| --- | --- | --- | --- | --- | --- | --- |
| *E. coli* | PE^+^/FITC^+^ (%) | 15.7 | 7.9 | 9.2 | 7.6 | 10.4 |
|  | PE^+^/FITC^-^ (%) | 12.7 | 18.9 | 16.2 | 17.4 | 10.8 |
|  | PE^-^/FITC^+^ (%) | 15.4 | 20.7 | 15.1 | 19.8 | 17.9 |
| *V. splendidus* | PE^+^/FITC^+^ (%) | 3.8 | 7.9 | 9.4 | 5.7 | 4.1 |
|  | PE^+^/FITC^-^ (%) | 20.6 | 16.8 | 14.7 | 17.5 | 15.2 |
|  | PE^-^/FITC^+^ (%) | 7.5 | 15.4 | 9.2 | 12.3 | 11.8 |
| *B. subtilis* | PE^+^/FITC^+^ (%) | 5.8 | 9.2 | 8.4 | 7.8 | 7.1 |
|  | PE^+^/FITC^-^ (%) | 7.8 | 15.6 | 11.2 | 13.6 | 8.9 |
|  | PE^-^/FITC^+^ (%) | 18.9 | 12.2 | 16.7 | 19.5 | 14.8 |
| *S. aureus* | PE^+^/FITC^+^ (%) | 9.5 | 8.6 | 5.2 | 7.8 | 6.4 |
|  | PE^+^/FITC^-^ (%) | 19.3 | 16.7 | 12.1 | 15.4 | 11.9 |
|  | PE^-^/FITC^+^ (%) | 15.4 | 18.8 | 19.2 | 11.7 | 17.6 |

*Lycopersicon esculentum* lectin staining

| Microbes | Fluorescence | Sample 1 | Sample 2 | Sample 3 | Sample 4 | Sample 5 |
| --- | --- | --- | --- | --- | --- | --- |
| *E. coli* | PE^+^/FITC^+^ (%) | 24.9 | 24.8 | 19.4 | 21.6 | 25.5 |
|  | PE^+^/FITC^-^ (%) | 3.9 | 4.3 | 2.5 | 3.1 | 2.2 |
|  | PE^-^/FITC^+^ (%) | 3.6 | 2.7 | 1.9 | 1.5 | 2.4 |
| *V. splendidus* | PE^+^/FITC^+^ (%) | 25.6 | 20.1 | 15.1 | 17.6 | 15.3 |
|  | PE^+^/FITC^-^ (%) | 2.7 | 5.6 | 4.3 | 3.8 | 4.2 |
|  | PE^-^/FITC^+^ (%) | 1.8 | 2.6 | 1.4 | 2.3 | 2.7 |
| *B. subtilis* | PE^+^/FITC^+^ (%) | 22.4 | 21.6 | 29.5 | 27.6 | 19.7 |
|  | PE^+^/FITC^-^ (%) | 1.9 | 3.8 | 2.1 | 2.9 | 3.0 |
|  | PE^-^/FITC^+^ (%) | 0.9 | 2.8 | 1.6 | 1.4 | 2.3 |
| *S. aureus* | PE^+^/FITC^+^ (%) | 22.7 | 25.8 | 34.1 | 25.4 | 30.3 |
|  | PE^+^/FITC^-^ (%) | 3.9 | 4.6 | 2.3 | 3.3 | 2.9 |
|  | PE^-^/FITC^+^ (%) | 1.8 | 0.7 | 2.6 | 2.4 | 2.7 |
